# Extraosseous Intra-Articular Osteochondroma

**DOI:** 10.1155/2013/181862

**Published:** 2013-09-19

**Authors:** Pragash Mohanen, Kumaresan Palania Pillai, Kanagasabai Rangasamy

**Affiliations:** ^1^Pondicherry Institute of Medical Sciences, 405 D Block, Srinivas Towers, Azeez Nagar, Reddiarpalayam, Pondicherry 605 010, India; ^2^Department of Orthopaedics, Sri Manakula Vinayagar Medical College and Hospital, Pondicherry 605 107, India; ^3^Department of Orthopaedics, Pondicherry Institute of Medical Sciences, Pondicherry 605 014, India; ^4^Sanjeevi Hospital, Kallakurichi, Tamil Nadu 606 202, India

## Abstract

*Background*. Conventional osteochondromas are common bone lesions developing in the metaphyseal region of growing skeleton. Marginal excision is the treatment of choice for such tumours. Extraosseous cartilaginous tumours are rare and their biological potential is poorly characterized. *Case Presentation*. A-52-year old woman presented with 3-year history of fullness and dull pain and inability to flex her left knee, sit cross-legged, or squat. Clinical and imaging studies revealed a nodular mineralised mass in the anterior portion of the knee displacing the patellar tendon laterally. Excision biopsy confirmed the diagnosis of extraosseous osteochondroma-like soft tissue mass. There is no recurrence at two-year followup. *Conclusion*. An integrated clinicopathological diagnosis helps to clarify the nature of extraosseous cartilaginous tumour that can arise at an unusual anatomic site. Complete surgical excision is the treatment of choice.

## 1. Background

Conventional osteochondromas are common bone lesions developing in relation to the periosteum and occur around the growth plate of long bones, especially the knee [1]. They tend to grow away from the joint. These tumours usually stop growing with closure of the physeal plate [1]. Conventional treatment is by marginal excision.

Synovial chondromatosis is a less-common proliferation of multiple nodules of hyaline cartilage within the synovium, in most cases requiring thorough synovectomy [2].

Para-articular or extraosseous osteochondromas are rare in older individuals [3] and their biological potential is poorly characterized. In joints with a large capsular space such as the knee joint, osteochondromas can remain intra-articular [4]. Usually arising in juxta-articular soft tissue without attachment to bone, these lesions may be very large and show histologic features suggestive of a malignant process, including hypercellularity of the cartilaginous component and atypia of individual chondrocytes. Furthermore, some of these tumours continue to grow after skeletal maturity. Because marginal excision is adequate management, it is important to distinguish the extraosseous osteochondroma from chondrosarcoma and synovial chondromatosis. The terminology used to describe these osteocartilaginous lesions has been historically inconsistent and confusing and so far only 21 cases have been reported in the literature [4–14] especially from the West.

Here we report one more case of extraosseous osteochondroma-like soft tissue mass in the anterior portion of the knee joint.

## 2. Case Report

A-52-year old woman presented with 3-year history of fullness and dull pain involving the left knee. She had been aware of a mass in the knee joint that had been progressively increasing in size. She was unable to flex her knee, sit cross-legged, or squat. There was no history of trauma or constitutional symptoms.

**Figure 1:**
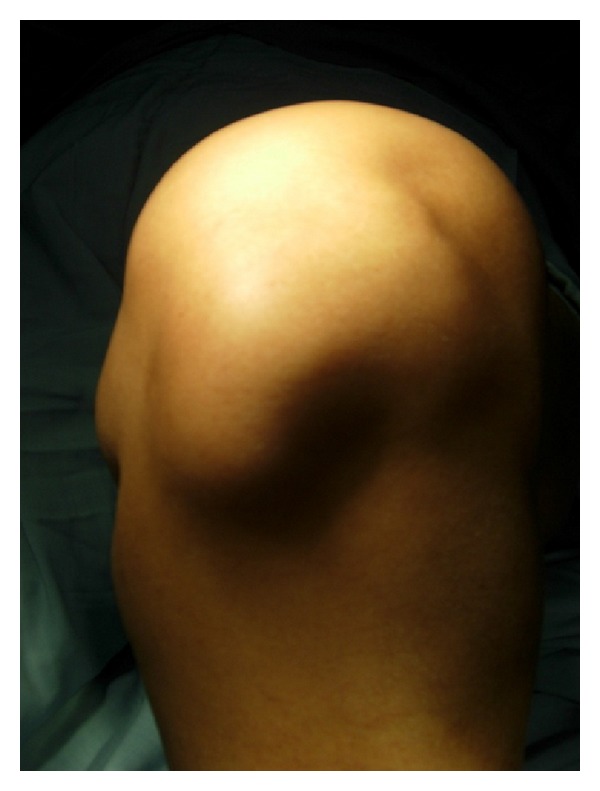
Clinical photograph.

**Figure 2:**
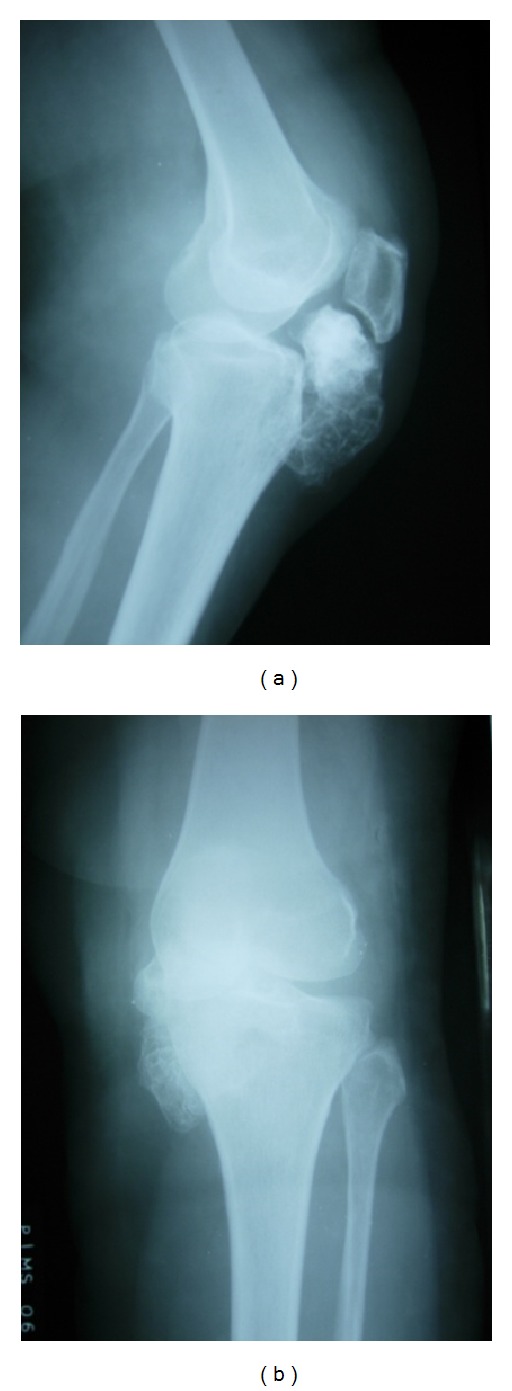
Preoperative X-ray.

**Figure 3:**
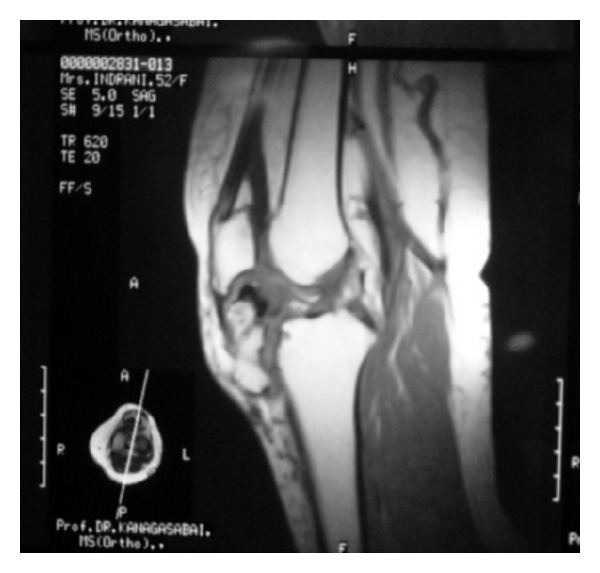
MRI of left knee.

On physical examination, there were fullness on the medial aspect of left knee and palpable nodular mass of approximately 6 × 4 cm over the infrapatellar region medial to the patellar tendon (Figure 1). The mass was nontender with no local warmth, bony hard in consistency, and immobile. The mass was more prominent on flexion than on extension of knee. The range of motion of the knee joint was limited to 100° of active flexion. Neurovascular examination was normal. Plain radiographs of the knee revealed a mineralized mass situated between the anterior tibial tubercle and the patella. The mass appeared to occupy the entire region of the infrapatellar fat pad. The femoral condyles and tibial plateau were normal (Figures 2(a) and 2(b)). Subsequently an MRI was performed. MRI revealed a large mineralized mass lying in the Hoffa body with no attachment to the skeletal tissue (Figure 3). It was decided to perform an excision biopsy.

**Figure 4:**
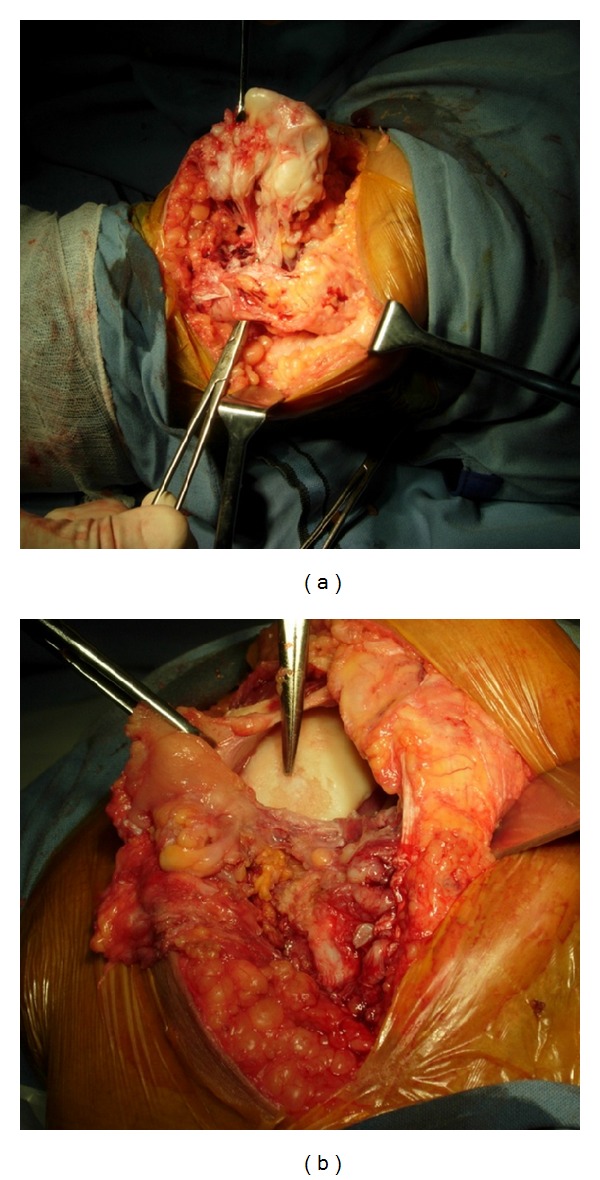
Intraoperative findings. (a) Tumour medial to the patellar tendon and (b) tumour impaling medial femoral condyle.

**Figure 5:**
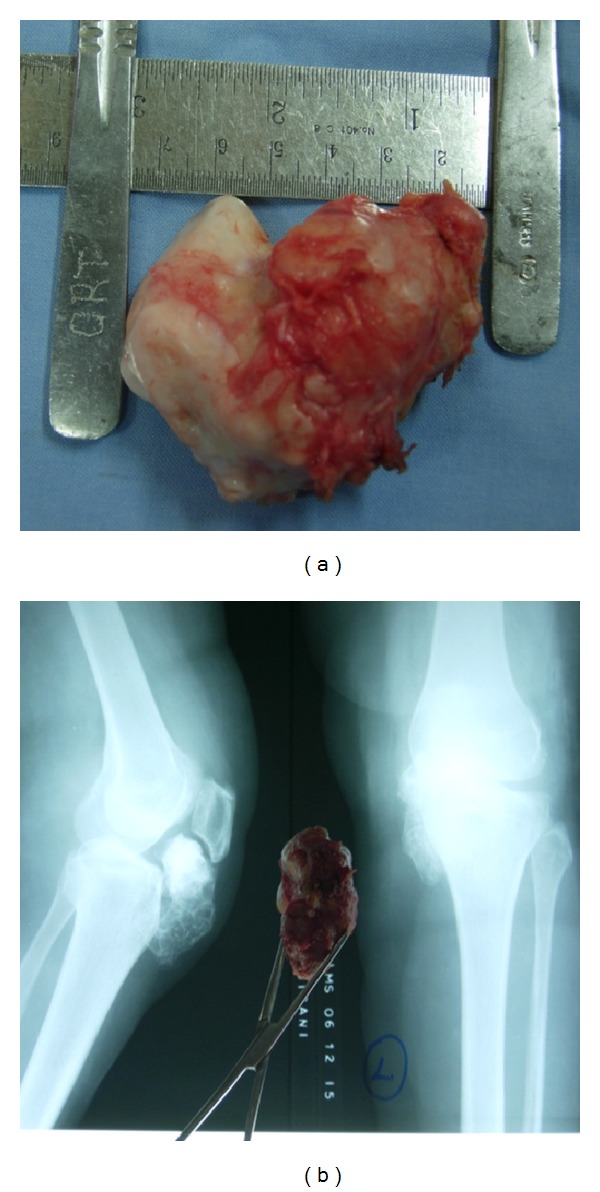
Excised mass in toto.

Under regional anaesthesia with the patient supine in a bloodless field furnished by a pneumatic tourniquet, a 7 cm longitudinal incision was made medial to the patellar tendon. The mass was lying medially and behind the patellar tendon in Hoffa's fat pushing the tendon laterally (Figure 4(a)) and impinging on medial femoral condyle and intercondylar notch (Figure 4(b)). It was dissected and excised in toto (Figures 5(a) and 5(b)). The medial tibial plateau, patella, menisci, and the patellar tendon were not involved in the process. The mass did not involve the joint space or synovium and was not continuous with femur, tibia, or patella but rather originated from joint capsule and was completely intra-articular. The capsule and the subcutaneous tissue were sutured with 1/0 vicryl and skin with 2/0 ethilon. 12 G drain was kept. Compression dressing was applied. Weight bearing was allowed in the immediate postoperative period.

Postoperative recovery was uneventful. Sutures were removed on the 12th postoperative period. The patient returned to her daily activities regaining full flexion within two weeks after surgery. She was on regular followup. When last reviewed 24 months after excision, she was asymptomatic with no clinical and radiographic signs of recurrence of the lesion (Figures 7(a), 7(b)(1), and 7(b)(2)).

**Figure 6:**
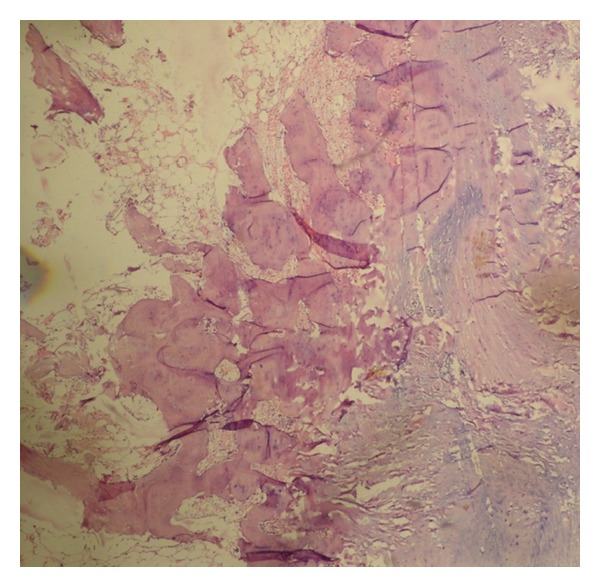
Histopathology: This figure shows bone marrow, bone and cartilage with fibrofatty tissue at the periphery.

**Figure 7:**
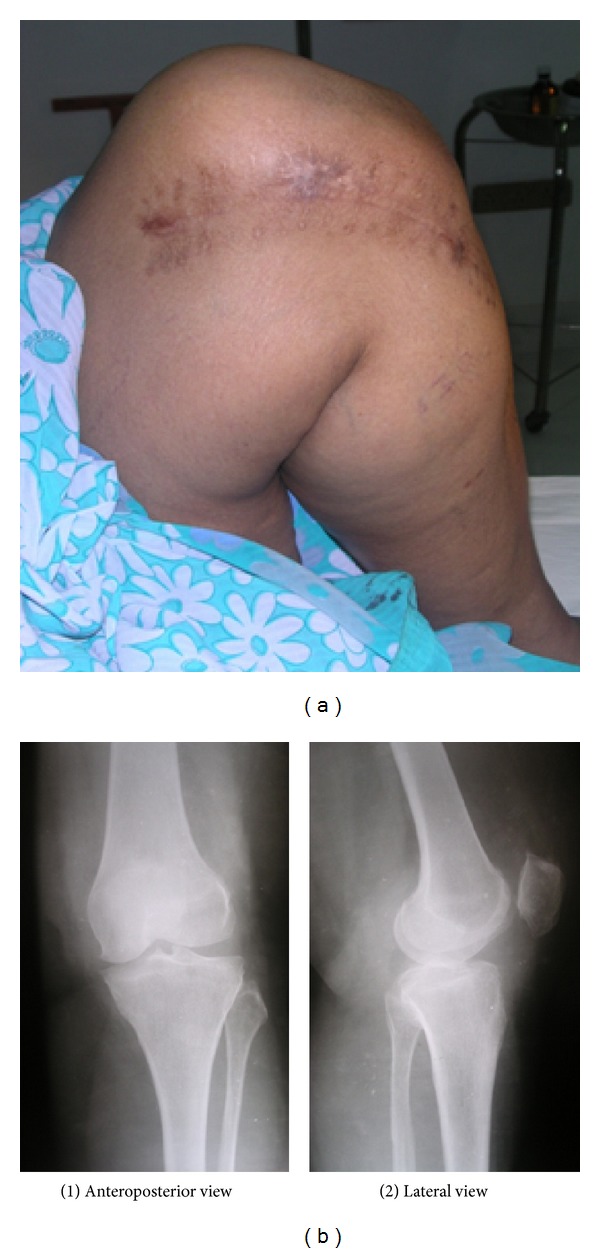
Two-year followup. (a) Clinical photograph and (b) radiology showing no recurrence.

Grossly, the resected specimen measured 7 × 6 × 5 cm with a large cartilaginous cap and was surrounded by adipose tissue and a fibrous capsule. Histologically, the cut sections showed numerous lamellar bony trabeculae with mature cartilaginous cap. Fibrofatty tissue was seen at the periphery (Figure 6). There were foci of active endochondral ossification without any evidence of malignancy. No mitotic figures were seen.

## 3. Discussion

There are several different types of lesions composed of bone and cartilage that occur around joints. A conventional osteochondroma arises from a developmental defect in the growth plate, resulting in an osteocartilaginous proliferation in which the bony stalk, continuous with that of the bone of origin, typically grows away from the nearest joint [15, 16]. Most osteochondromas involve the knee region although they may develop in any bone that forms by endochondral ossification. Microscopically, the cartilage cap of an osteochondroma may show histologic features of proliferation until the patient reaches skeletal maturity, at which time growth of the osteochondroma and proliferation of the cartilage should cease.

Extraosseous osteochondromatous lesions are rare. Usually arising from the joint capsule or para-articular soft tissue without attachment to bone, these lesions may be large with clinical and radiological features of malignant process. These have been reported earlier as para-articular osteochondroma, intracapsular chondroma, intra-articular osteochondroma, extraosseous osteochondroma, soft tissue osteochondroma, capsular osteoma, ossification of infrapatellar fat pad, and ossifying chondroma [4–14].

The concept of para-articular osteochondroma was first introduced in 1958 by Jaffe [6], who used the synonymous terms para-articular osteochondroma and intracapsular chondroma to describe osteochondral metaplasia occurring in the fibrous joint capsule or soft tissue adjacent to a joint [6]. Milgram and Dunn [4] were the first to use the term para-articular osteochondroma and to differentiate the same lesion from synovial osteochondromatosis.

Only few intra-articular osteochondromas have involved the anterior and more rarely the posterior knee joint space [1, 4, 9, 14]. Bleshman and Levy reported an intra-articular osteochondroma of the hip with lateral displacement of femoral head [3]. In our patient, plain radiographs showed a large, well-circumscribed, and mineralized mass without abnormal calcifications within the adjacent tissue.

At MRI, there were no irregularities or thickening of the cartilaginous cap greater than 1 cm. Hence, there was no suggestion of malignant feature [17]. The borders of the mass were well defined displacing the patellar tendon and the Hoffa fat without infiltration [17]. The size of the lesion and small areas of chondroid tissue made synovial chondromatosis unlikely. Malignant degeneration to chondrosarcoma has to be considered under differential diagnosis as the patient attained skeletal maturity, and proliferation of cartilage should have ceased by that time [1, 5, 17, 18].

Intraoperatively, the tumour was completely intra-articular with no evidence of continuity to bone. The gross appearance and histological examination demonstrated the features of an extraosseous osteochondroma-like soft tissue mass with secondary bone formation similar to normal enchondral growth [18]. The benign course following the removal with no recurrence at 2 years is in accordance with various reports [4, 8, 9, 18].

MRI is recommended for further characterization of nature and extent of an intra-articular osteochondroma. Operative removal is the procedure of choice when function is reduced and nature of tumour is uncertain.

In conclusion, an integrated clinicopathological diagnosis helps to clarify the nature of extraosseous cartilaginous tumour that can arise at unusual anatomic site. Complete local surgical excision is the treatment of choice.
